# The Organization of National and Local Forces in the Campaign against Cancer

**Published:** 1915-06

**Authors:** Curtis E. Lakeman

**Affiliations:** Executive Secretary, American Society for Control of Cancer


### THE ORGANIZATION OF NATIONAL AND LOCAL FORCES IN THE CAMPAIGN AGAINST CANCER.

By Curtis E. Lakeman, Executive Secretary, American Society for Control of Cancer.

The American Society for the Control of Cancer has recently urged that every state medical society take an active part in arranging meetings and in spreading among all members of the profession the latest knowledge of malignant disease. At the suggestion of the Concer Committee of the Pennsylvania

State Medical Society, many journals will devote their July issues to this subject. It has been pointed out that the American Society for the Control of Cancer might take this timely opportunity to state its view of the relations between the various bodies which are concerned in this campaign. The suggestion is welcome. If indeed a clear understanding can be reached as to the most effective division of functions and duties among the various organizations, national, state and local, interested in this subject, a long step will have been taken toward the conquest of malignant disease, in so far as that ideal can be approached by the practical application of present knowledge.

### The National Society.

The American Society for the Control of Cancer sets up no claim of priority or originality in preaching to the public the necessity of early recognition and treatment of this disease. The organization was effected under the inspiration of numerous similar movements in this country and in Europe. From the first it has been inspired only by a sincere ambition to co-ordinate all existing forces into a single irresistable nation-wide effort to reduce the cancer death rate by imparting the necessary knowledge and inspiring the will to believe and act upon it. Those who direct the policy of the Society have no illusions that they are "called" above others to this task. They firmly believe that all sincere workers should unite in a single well considered national movement. If the present Society fails to meet the requirements of such a movement it must give place to some agency that will do so, leading the campaign against malignant disease with as conspicuous ability and success as the National Association for the Study and Prevention of Tuberculosis has directed the war on consumption.

### Relation to the Professional Societies.

While the Cancer Society found its first impulse in the work of a committee of the American Gynecological Society, the movement was broadened at its very inception by the appointment of organizing delegates from the American Surgical

Association, the American Dermatological Association, the Association of Pathologists and Bacteriologists and practically all the similar special organizations which met in Washington in May, 1913, as the Congress of American Physicians and Surgeons. Definitely launched in New York on May 22nd, 1913, the movement received within a few months the official endorsement of the American Medical Association, the Clinical Congress of Surgeons, the Western and Southern Surgical and Gynecological Societies and a number of sectional and state organizations. All these professional bodies have endorsed the design of the National Cancer Society as expressed in its Constitution:

"To disseminate knowledge concerning the symptoms, diagnosis, treatment and prevention of cancer, to investigate the conditions under which cancer is found and to compile statistics in regard thereto."

### Relation to Cancer Research.

It will be seen that this purpose comprises not only the conduct of an educational campaign, but the gathering of information in regard to this disease. In what relation, then, does the Society stand to the various American Cancer research institutions and workers? The answer is that the Society does not contemplate the prosecution or support of biological research, already so ably pursued under the auspices of our leading universities. With these workers in the field of pure science mutually helpful relations have developed. Indeed a notable collective expression of their attitude is regarded as a very corner stone of the educational movement. A few years ago the eminent laboratory students placed on record in the transactions of their official organization, the American Association for Cancer Research, their conviction that pending the discovery of the ultimate nature and cause of cancer, a far more effective dissemination and utilization of the vast store of present knowledge of the disease is urgently called for. Formed to carry out this very object the "Control" Society depends upon the constant support and co-operation of the institutions represented in the "Research" Society. Many of the foremost

American students of cancer are prominent in the membership of both organizations. Machinery is thus provided for the wider dissemination among the profession and the people of the essence of the newest knowledge of malignant disease, fresh from its laboratory sources.

### Relation to Statistical Investigations.

The Society does, however, contemplate original work in the collection and collation of statistical data, and will expand this feature of its program as fast as its resources permit. The statistics of cancer mortality need to be improved both as regards their collection and their publication. The merest suggestion by the Society to the United States Census Bureau has been sufficient to initiate a notable advance in this respect. With the greatest possible interest and zeal, Mr. Harris, the late Director of the Census, and his successor, Mr. Rogers, have undertaken the preparation of a special report on the cancer mortality of the United States Registration Area in 1914. The number of deaths will be stated in full detail under some thirty titles of organs and parts of the body affected, instead of, as hitherto, merely under the six general groups of the International List. The Imperial Cancer Research Fund has long urged that it is only on the basis of such detailed data for the various organs that a true conclusion can be reached as to whether or not cancer is increasing. For the first time in the United States the data will now be at hand, as it is in England and Wales through the reports of the Registrar-General, for the prosecution of such inquiries.

The Census Bureau will also for the first time in this study make a distinction between returns based on certain and on doubtful diagnosis. To secure the additional information needed for this distinction the Bureau is sending tens of thousands of letters to physicians who have certified deaths from cancer asking whether the diagnosis was based on clinical findings alone or was established by surgical intervention, microscopical examination, or autopsy.

All this, it will be realized, is a large amount of work for even a government bureau to undertake. Much of it should be done in the first place by the registration offices and the boards of health of the several states, where the original certificates of death are filed. It will be the duty of the American Society for the Control of Cancer to urge upon the various state officials the need of undertaking this work in order to insure the permanence of the advance in the statistical study of cancer which has been inaugurated by the Census Bureau.

But the Society is also interested in special statistical studies of the geographical, racial and occupational distribution of cancer, and above all in collating, upon a uniform plan, the records of surgical treatment of the disease in the leading hospitals. It is important that an authoritative answer be available for all who ask just what percentage of success is to be expected in the treatment of each phase and each stage of this multiform disease. All such studies the Society regards as fulfilling its fundamental purpose and in pursuing them it is everywhere receiving the most cordial encouragement and assistance from statistical offices and from the best hospitals and institutions.

### Relation to Educational Agencies.

The important and clearly established lessons derived from such studies of the sources of information must be given to the public. The Society has undertaken to do this indirectly, through its publications, its regular articles for the newspapers and its lectures. But in the large view it can best secure this object by enlisting the co-operation of all appropriate existing agencies which conduct educational work. Foremost among these are the state and local departments of health, especially those which are devoting an increasing share of their energies to the spreading of the gospel of health by bulletins, exhibits and lectures. In the same category must be included the committees on public instruction which in many states are conducting admirable campaigns of health education under the auspices of the state medical societies. Toward all these agencies the Society stands in the relation of the "producing" to the "distributing" end of a manufacturing business. With its wide outlook over the national field it is in a strong position to provide statistical material, to receive and pass on new knowledge, new experiences, new methods which have been found valuable in one field and should be used in others. In another view the Society may take the position of "middleman" between the research workers and statistical students producing new facts about cancer at the sources of knowledge on the one hand, and on the other the many agencies, general and local, which will bring the practical bearings of this knowledge, new and old, directly home to the people. In general, then, one of the most useful functions of the Society is to act as a bureau of information and clearing house which is at the service of all workers and institutions interested in the study and control of cancer.

### Relation to State Committees.

The relation of the national Society to similar movements within the various states should be clear from what has been said. In no case will the Society seek to set up local agencies to parallel work already adequately organized under the auspices of state medical societies and boards of health. Provision is made for local committees to be organized under the supervision of the resident directors of the National Society wherever no state or local agency is in a position to undertake the work. Such groups will not be formed, however, except under full agreement with present state agencies. Where, as in Pennsylvania, under Dr. Wainwright, and similarly under the auspices of state medical societies in Maine, Wisconsin, Kansas, Colorado, Louisiana, Texas and many other states, active local committees are at work, every effort will be made to assist these groups in the manner already outlined and so far as the constitutional limits of size permit to secure from them representative delegates to the governing council of the National Society. At least one director from each state will eventually be chosen to act as a local correspondent who will inspire and stimulate work in his own state while at the same time assisting in formulating the general policies of the National Society.

### Relation to Other General Committees.

It is an earnest of the good feeling and harmony with which the cancer campaign is evolving toward a single coherent national movement to consider the high degree of integration with other national agencies which has already been attained. Some of these had begun effective work long before the present Society was established. Aside from such admirable local campaigns as that of the Pennsylvania Commission and the work inspired by Dr. C. C. Carstens in Michigan, the Clinical Congress of Surgeons of North America had in the field an active Committee on Cancer under the chairmanship of Dr. Thomas S. Cullen of Baltimore, the other members being Dr. Simpson, of Pittsburgh, and Dr. Howard C. Taylor, of New York. This Committee, as is well known caused the publication of widely read and influential popular articles by Samuel Hopkins Adams. It is instanced merely as indicative of the get-together spirit that animates the National Society that all three of these men naturally took their places as members of the Executive Council of the new association. Subsequently the American Medical Association appointed a Cancer Committee representing its Council on Health and Public Instruction, and again to avoid duplication of effort the same men were made members of that Committee. Dr. Frederick R. Green, the capable executive of this Council of the American Medical Association, has been from the first a director of the Cancer Society, and has given invaluable advice and co-operation in its publicity campaign, printing every week in the press bulletin of the A. M. A. a popular article on cancer prepared by the Society, which thereby reaches 3,000 or more editors in all parts of the country.

A similar identity of committees has been effected in local fields, especially in Minnesota, and is typical of the desire to carry on everywhere a well-co-ordinated national campaign which shall embrace representation from all the principal local agencies and shall thus move forward with absolute harmony and unity of purpose to the accomplishment of its difficult but glorious ideal—the progressive reduction of the mortality from this historic scourge of mankind.

